# Toxic Habits and Well-Being Measures in Spanish Healthcare University Students during the COVID-19 Pandemic

**DOI:** 10.3390/ijerph192013213

**Published:** 2022-10-14

**Authors:** Irene Zapata, José Luis Maté-Muñoz, Alfonso Higueras, Juan Hernández-Lougedo, Natalia Martín-Fidalgo, Pablo García-Fernández, María Victoria Redondo-Vega, Jaime Ruiz-Tovar

**Affiliations:** 1 Department of Medicine, Alfonso X El Sabio University, 28691 Madrid, Spain; 2 Department of Radiology, Rehabilitation and Physiotherapy, Complutense University of Madrid, 28040 Madrid, Spain; 3 Independent Researcher, 28691 Madrid, Spain; 4 Department of Physical Activity and Sports Science, Alfonso X El Sabio University, 28691 Madrid, Spain; 5 IdISSC, Instituto de Investigación Sanitaria del Hospital Clínico San Carlos, 28040 Madrid, Spain

**Keywords:** alcohol, smoking, cannabis, toxic habits, stress, sleep, self-perceived health, COVID-19 pandemic, university students, healthcare students

## Abstract

Background: Unhealthy lifestyles are strongly entrenched in healthcare universities and have sometimes been linked to stress or lack of sleep. This study investigated the prevalence of toxic habits (smoking, patterns of harmful alcohol use, and illicit drug use), stress levels, perceived health status, and sleep duration and assessed the connections between toxic habits and said well-being measures, as well as healthcare students’ perception of the influence of the COVID-19 pandemic on these health-related behaviors. Methods: In a cross-sectional study, healthcare students from Alfonso X University (Spain) completed a health survey composed of Alcohol Use Disorders Identification Test (AUDIT-C), Perceived Stress Scale (PSS-10), self-perceived health status, and the number of hours of sleep. Results: A total of 997 healthcare students completed the survey, of which 982 were analyzed. Being a smoker (32.2%) was associated with worse health status and insufficient sleep. Risk drinkers (33.2%) were associated with being female, and the consumption of cannabinoids (6.7%), with being male. These three toxic habits were related to each other. High levels of stress (28.2%) were correlated with worse ratings in the perception of health status (29.2%) and with insufficient sleep (45.8%), and all of them were associated with the female sex. Respectively, 49.3% and 44.2% of students recognized a worsening in their perception of stress and their sleep habits during the pandemic. Conclusion: Healthcare universities must carry out health promotion programs for stress management, sleep habits, and unhealthy lifestyles.

## 1. Introduction

Numerous studies have linked unhealthy habits, such as smoking, alcohol, or other drug use, with the development of non-communicable diseases, such as cardiovascular, renal or respiratory diseases, diabetes, cancer, and dementia, all of which are responsible for 70% of deaths worldwide [1,2].

Preventing these modifiable risk factors in the general population is the direct responsibility of healthcare professionals [3,4], and they should be adequately trained to do so.

We must consider that future healthcare providers are university students who are mostly in the stage of adolescence or early adulthood, where important milestones occur, such as leaving the family home, increased individual autonomy, and a greater desire to experience sensations and be accepted in their peer group. Thus, they are a vulnerable population for the acquisition of unhealthy behaviors [5] and could partly explain the high prevalence of smoking, alcohol, and illicit drug use among healthcare students. For example, Tavolacci et al. [6], in a sample of more than 1000 healthcare students, found that one out of four students was an active smoker, used alcohol frequently, or had used psychotropic drugs some time in their lives. In addition, it was found that the use of all these harmful substances tended to cluster at times among university students who used them [7,8,9].

Knowing that the lifestyles that are acquired in this stage will likely drastically influence those that develop during adulthood [10,11] and that, in turn, there is a close relationship between the healthy behaviors of healthcare professionals and their positive attitude towards health promotion [12,13,14], it is essential to focus our attention on the acquisition of a healthy lifestyle by these university students in the field of healthcare.

In addition to harmful substances, two other determinants that directly influence the health of the college population are stress and sleep duration [15]. Severe stress in the higher education stage may be caused by high academic demands, along with changes in the life situations of this population [16]. In addition, healthcare students must learn to cope with illness and death in their clinical practice [17].

Stress in the university population is associated with poorer physical health, poor academic performance, and numerous mental illnesses, such as depression, anxiety, anorexia, or substance abuse, among those who suffer from it [18]. On the other hand, a large number of studies found a strong association between higher levels of stress and specific groups of university students, such as female students [19,20]. For all these reasons, the investigation of stress in university students is transcendental.

In addition to being related to health, the stress in the university population was correlated by some authors with the consumption of toxic substances, objectifying that the most stressed university students presented a higher prevalence of smoking or alcohol abuse [6,19,21]. On the other hand, other authors found no relationships between stress and smoking, alcohol use [22,23], or illegal drugs [7].

Another important measure related to well-being and health is sleep duration, whose alteration is related to different physical and psychiatric illnesses [24]. A sleep duration of 7 h is considered optimal, as it is associated with better cognitive function and mental health [24]. Some studies showed high rates of sleep problems among university students and reported a relationship between these difficulties, and severe stress levels [25] and higher rates of smoking [26], without finding an association with the consumption of other substances, such as alcohol [25]. Therefore, as in the case of stress, there is an association between toxic habits and this measure of well-being that must be clarified.

Since the moment when the WHO declared COVID-19 infection a global pandemic in March 2020, its consequences have been objectified in all spheres of society, including changes in lifestyles and increased levels of stress, both in the general population [27,28] and especially in vulnerable groups, such as healthcare workers, who suffered high rates of stress, depression, and anxiety [29,30]. This shows that it should be a priority to train future healthcare workers in stress management and in the adoption of healthy habits to prevent possible burnout related to patient care in this extraordinary situation [31].

Another group vulnerable to worsening well-being during the pandemic is university students [32], specifically healthcare students, who were found to experience severe stress rates during the first phase, i.e., the period of home confinement [33,34]. On the other hand, there are fewer studies on this issue in the second year of the pandemic, i.e., when a relative normalization of the population’s daily life was achieved, although still coexisting with lifestyle changes compared with the pre-pandemic period, marked according to the epidemiological situation, such as the use of masks, restrictive measures about leisure activities or travel, or in the university context, home isolation of classrooms depending on the number of people infected, with a shift to online classes, among others.

It has also been studied how the pandemic caused changes in drinking habits in the general population. In a large European study carried out during the first months of the pandemic, a decrease in alcohol consumption in the adult population was observed in 19 of the 21 European countries investigated [35]. In contrast, in systematic reviews and meta-analyses published in the same period, no significant changes were found in the consumption of alcohol or smoking in the general population despite a higher number of attempts to quit both substances [36,37].

We also found contradictory results in the university population. Authors such as Du et al. [38] and Jaffe et al. [39] found a decrease in alcohol consumption among their students during confinement, while other authors found no changes in this harmful habit among their students during this period [40]. A continuous evaluation of the impact of the pandemic on the evolution of health determinants is necessary [27,40], and few studies analyzed whether there have been changes in the consumption of toxic substances, as well as their relationship with stress, sleep, or health, during the second year of the pandemic.

The aim of this study was to define the prevalence of toxic habits (smoking, harmful alcohol use patterns, and illicit drug use), stress levels, perceived health status, and sleep duration among healthcare university students, as well as to evaluate the connections between toxic habits and said well-being measures. Finally, we wanted to investigate the students’ perception of the influence of the COVID-19 pandemic on these health-related behaviors.

## 2. Materials and Methods

### 2.1. Study Design

A cross-sectional unicentral study was carried out on a population of university students with degrees related to healthcare at Alfonso X el Sabio University (UAX) in Madrid (Spain).

### 2.2. Sample and Procedure

Non-probabilistic convenience sampling was used in this investigation. The recruitment criteria were students aged at least 18 years and registered during the academic year of 2021–2022 in one of the academic careers related to healthcare offered by UAX (Medicine, Nursing, Physical therapy, Biomedicine, Physical activity and sport sciences, or Biomedical Engineering). Data were collected between November and December 2021.

This study was conducted through an anonymous survey that was self-completed on an online platform by the student when attending an in-person class. The researchers informed the participants about the study’s objectives and the confidentiality that would be maintained in the data collected throughout the study. Their participation was voluntary, and they did not receive any reward for participating.

### 2.3. Ethical Considerations

The study was conducted following the Declaration of Helsinki, and the protocol was approved by the Clinical Research Bioethics Committee of Alfonso X el Sabio University (Resolution 2021_10/099). All participants gave their informed consent before participation, knowing that they could revoke their collaboration during the investigation.

### 2.4. Measures

The survey (Annex 1) was based on the Spanish National Health Survey [41], in association with several questionnaires and validated definitions [42,43,44,45,46,47], and it was divided into 3 main parts: demographic characteristics, toxic habits, and well-being measures (stress, health status, and sleep habits). Thus, the parameters that were examined were those detailed in the next subsections.

#### 2.4.1. Demographic Characteristics

The survey included age, sex, academic major and year, and diverse socioeconomic features (financial support for the studies and cohabitation).

#### 2.4.2. Toxic Habits

Smoking habit: This was defined as non-smokers, active smokers, or former smokers, who were those that had remained abstinent for at least the last 6 months. For establishing risk ratios, the sample was aggregated into smokers and non-smokers. Smokers were also asked about their intention to quit the habit. Finally, exposure to tobacco smoke was also investigated.

Alcohol consumption: The alcoholic habit was determined according to the frequency of alcohol consumption, classifying the participants as habitual drinkers if they declared consuming alcoholic beverages at least once a week, occasional drinkers if their consumption was less than once a week, or non-drinkers.

The Alcohol Use Disorders Identification Test (AUDIT-C) was used to detect problems due to alcohol abuse; it consists of 3 questions on the consumption habit and is adapted from the longer AUDIT questionnaire [45]. The scores for each item can range from 0 to 4 points, resulting in a final score of 0 to 12. A score of >5 for men and >4 for women identifies people who are high-risk drinkers. It was created in the field of primary care and validated for university populations [46]. In addition, binge drinking was assessed, as defined by the intake of the equivalent of 60 g of pure ethanol or more on one occasion [47]. Based on the frequency of episodes, it was classified into 5 categories: daily or almost daily, weekly, monthly, less than once a month, or never. The first three categories were grouped as frequent binge drinking to establish risk ratios.

Drug consumption: It was quantified by the answer to the following question: “How often do you consume any of the following substances: Tranquilizers, cannabis, cocaine, ecstasy, hallucinogens, or amphetamines?” The possible answers were “never”, “occasionally”, “weekly”, or “daily” for each of two time periods (“throughout your life” and “within the last 30 days”), following those reported in other studies [21].

#### 2.4.3. Well-Being Measures

Stress perception: This was assessed using the Perceived Stress Scale-10 (PSS-10) [42], which was validated by Remor [48] for the European population. This self-report instrument assesses the level of perceived stress during the last month through 10 items with a Likert response format. The maximum score obtained was 40 points. Taking similar studies as a reference, the participants were categorized into three stress levels for comparison between groups: low (0–13 points); moderate (14–26 points); high (27–40 points) [18,49]. Subsequently, these results were reconverted to a STEN scale; scores below 4 points were considered low stress, and scores above 7 points were considered high stress. Cronbach’s alpha was used to analyze the internal consistency of the PSS-10 for the entire scale, and for the adjusted item–total correlations. Based on the results obtained (α = 0.85 (0.82 to 0.87); mean item–total correlations ≥ 0.635), no items were eliminated, as all substantially contributed to the scale.

Self-assessment of health status: This measure was used as a global health indicator [43] and was obtained via the answer to the question: “Within the last 12 months, how do you rate your overall health status?” The five possible answers were “very good”, “good”, “fair”, “bad”, or “very bad”. The participants were categorized into two groups for descriptive analysis; students in one group answered “fair”, “bad”, or “very bad”, and students in the other group answered “good” or “very good”, based on previous research [21]. This measure of health, despite being collected through a single item, has been related in numerous studies to mortality and the components of subjective assessment of health [43,50].

Sleep duration: This was also assessed by determining the mean number of hours of sleep, considering an insufficient sleep duration when it lasted less than 7 h, as defined by other authors [24,51].

#### 2.4.4. Perception of the Influence of the COVID-19 Pandemic on the Variables Described

As Du et al. [38] investigated at the beginning of the pandemic, the participants were asked about their perception of changes in toxic habits and measures of well-being investigated through questions such as “Comparing your current state of health with the one you had before the COVID-19 pandemic, you would say that…”, with possible answers including: “my state of health is now the same”, “my state of health is now better”, and “my state of health is now worse”. Regarding sleep, the students were asked subjectively about the possible change in sleep habits.

### 2.5. Statistical Analysis

Data analysis was performed using SPSS Version 22.0 (SPSS Inc., Chicago, IL, USA).

Quantitative variables following normal distribution were defined using mean and standard deviation. Discrete variables were defined using some cases and percentages.

For the comparison between qualitative variables, the chi-square test was used. When the expected value was less than 5 in any of the boxes of the contingency table, it was necessary to use Fisher’s exact test. The magnitude of the association was estimated using the Relative Risk (RR) ratio and expressed with a 95% Confidence Interval (CI). For the comparison of independent quantitative variables, Student’s *t*-test and the ANOVA test were used. A *p*-value < 0.05 was considered statistically significant.

## 3. Results

### 3.1. Demographic Characteristics

The total population consisted of 2059 students, of whom 997 gave their consent to participate in this research study and completed the questionnaire. Fifteen participants were excluded due to incomplete surveys. Thus, data from 982 students were analyzed, including 652 females (66.3%) and 330 males (33.7%), with a mean age of 20.5 ± 3.8 years. Of the total sample, 854 (86.9%) were Spanish students, and 128 (13.1%) were foreign students. The academic major of the students and the years are described in Table 1. Most students belonged to the Medicine and Nursing degrees, and 43.5% of the participants were in their first year of university. 

The socioeconomic features of the sample are described in Table 1. Remarkably, 42.3% of the subjects lived with their families, and 88% of them recognized that the university costs were supported by their family income.

### 3.2. Toxic Habits

#### 3.2.1. Smoking Habit

A total of 64.3% of participants were non-smokers, while 32.2% (316) were active smokers, and 3.6% were former smokers. Of the active smokers, 120 (38%) failed an attempt to quit smoking, whereas 286 (90.6%) seriously intend to quit in the next months. Of the total sample, 20.1%, 28.7%, and 68.7% were exposed to tobacco smoke at home, inside the university campus, and in entertainment venues, respectively.

#### 3.2.2. Alcohol Intake

A total of 872 (88.7%) subjects drank alcoholic beverages, 57.8% were habitual drinkers, and 30.9% were occasional drinkers. Only 35.3% of the students reported drinking alcohol daily, whereas 74.1% drank alcoholic beverages during the weekend. According to the AUDIT-C classification, the sample’s prevalence of risky alcohol consumption was 33.2%, and 21.7% frequently binge-drank (at least monthly) (Table 2).

#### 3.2.3. Use of Drugs

In the last 30 days, students had consumed the following drugs at least once per week: 5.6% benzodiazepines, 6.7% cannabinoids, 0.5% cocaine, 0.3% ecstasy, 0.2% amphetamines, and 0.1% LSD. The frequency of drug intake is shown in the following table (Table 2).

#### 3.2.4. Associations among Toxic Habits

Dangerous alcohol intake, according to the AUDIT-C definition, was significantly associated with active smokers (RR, 2.93; 95% CI (2.17–3.96); *p* < 0.001). Similarly, active smokers were also associated with binge drinking habits (RR, 2.58; 95% CI (1.89–3.53); *p* < 0.001).

Active smokers were also significantly associated with cannabinoid use (RR, 3.69; 95% CI (2.58–5.26); *p* < 0.001), whereas benzodiazepines and other drugs did not show any significant association with the smoking habit.

**Figure 1:**
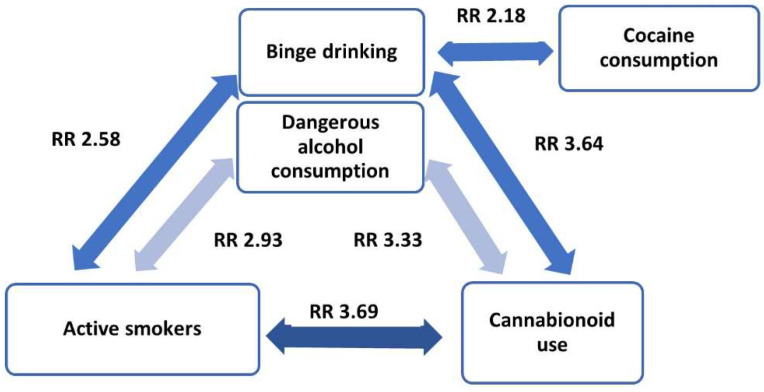
Relationships among toxic habits.

Binge drinking was associated with cannabinoids (RR, 3.64; 95% CI (2.52–5.25); *p* < 0.001) and cocaine (RR, 2.18; 95% CI (1.11–4.29); *p* = 0.027). In contrast, dangerous alcohol consumption, according to the AUDIT-C criteria, was only significantly associated with cannabinoid intake (RR, 3.33; 95% CI (2.33–4.77); *p* < 0.001) (Figure 1). Benzodiazepines and other legal drugs did not show any significant association with alcohol intake.

### 3.3. Well-Being Measures

A prevalence of stress perception was obtained and evaluated with the PSS-10, with 11.4% of the subjects presenting low stress, 60.4% medium stress, and 28.2% a high level of stress.

The perception of health status during the last 12 months is summarized in Table 3. A total of 29.2% of the sample considered it fair, bad, or very bad.

Regarding sleep duration, 448 participants (45.8%) acknowledged sleeping less than 7 h, which is considered insufficient sleep. The mean sleep time among all the students was 6.5 ± 2.3 h, with 46.5% of them perceiving it as restful sleep.

#### Associations among Well-Being Measures

Good–excellent health status perception was significantly associated with lower stress levels (PSS-10) (*p* < 0.001) and with sleep duration of at least 7 h/day (RR, 0.75; 95% CI (0.57–0.98); *p* = 0.041). Likewise, such sleep duration was also correlated with lower stress levels (*p* = 0.003).

### 3.4. Associations between Toxic Habits and Well-Being Measures

Students exposed to tobacco smoke at home reported a worse health status than those who were not exposed (RR, 1.429; 95% CI (1.027–1.989); *p* = 0.036).

Smokers were associated with insufficient sleep duration (<7 h/day) (RR, 1.47; 95% CI (1.12–1.92)) and poorer perceived health status (RR, 1.64; 95% CI (1.23–2.19); *p* = 0.001).

However, we failed to demonstrate an association between any toxic behavior analyzed with perceived stress, according to the PSS-10. We were also unable to demonstrate an association between the harmful use of alcohol or other drugs with sleep duration or perceived health status.

### 3.5. Influence of Sociodemographic Features on Health Behaviors

#### 3.5.1. Health Behaviors According to Gender

Despite the fact that 12.9% more females than males showed risky alcohol consumption (37.5% versus 24.6%, respectively; *p* < 0.001), there were no significant differences in the prevalence of binge drinking. In contrast, females showed a lower cannabinoid intake habit (RR, 0.60; 95% CI (0.42–0.85); *p* < 0.001). There were no differences in the rest of the toxic habits according to gender.

Females presented a higher stress perception than males, as assessed by the PSS-10 questionnaire (Table 4).

Simultaneously, females reported worse health status (RR, 1.9; 95% CI (1.392–2.594); *p* < 0.001) and insufficient sleep duration (RR, 1.53; 95% CI (1.17–2); *p* = 0.02) than males.

#### 3.5.2. Health Behaviors According to the Type of Academic Major

Nursing students presented a higher smoking habit than the rest of the students (RR, 1.86; 95% CI (1.414–2.454); *p* < 0.001) (Table 5).

Physical therapy students showed a higher frequency of binge drinking (RR, 2.48; 95% CI (1.59–3.88); *p* < 0.001) and high-risk drinkers (RR, 2.17; 95% CI (1.39–3.38); *p* < 0.001) than the rest of the university degrees analyzed.

### 3.6. Students’ Perception of the Influence of the COVID-19 Pandemic on Health Behaviors

Although most respondents (77.2%) considered that their smoking habits had not changed since the year before the COVID-19 pandemic, 2.6% acknowledged having started smoking during that period, and 9.4% said that they currently smoked more often. In contrast, 3.3% quit smoking, and 7.4% reduced their consumption (Table 6).

A total of 55% of the participants recognized that the COVID-19 pandemic did not alter their alcohol consumption, although 22.6% reported an increase in their alcohol intake.

When comparing the stress perception during the last 12 months with their previous stress perception before the pandemic, 49.2% of the participants recognized an increase during the pandemic period.

This was in turn correlated to the fact that 44.2% of the students identified that they began to sleep worse during the pandemic period.

However, similar proportions of students perceived their current health status to be worse (27.7%) and better (30.5%) than in the 12 months before the pandemic.

## 4. Discussion

The present study analyzed toxic habits and measures related to well-being in a population of Spanish healthcare students during the second year of the COVID-19 pandemic, when social distancing measures were reduced but still persisted and were constantly changing according to the evolution of the COVID-19 infection.

### 4.1. Toxic Habits and Well-Being Measures

#### 4.1.1. Toxic Habits

Almost one-third of the sample were active smokers, and two-thirds acknowledged exposure to tobacco smoke in entertainment venues. These figures were in line with studies of other university populations [52,53], although it is striking that nursing students had a higher frequency of smoking than students of other healthcare degrees (RR, 1.86; 95% CI (1.414–2.454); *p* < 0.001). This finding was already documented in studies conducted with healthcare students [6,54], as well as among healthcare professionals, with a higher prevalence of smoking among nurses than among physicians [55]. This also confirms that habits acquired in adolescence have a decisive influence on adult life and that they are perpetuated in many cases [11].

The significance of this finding does not only redound to the health of these young people but also to a likely worse predisposition to promote healthy habits, as was reported by Duaso et al. [56] in a study where smoking nurses were 25% less likely to organize a follow-up for smoking cessation. This fact was also found in studies of both smoking and non-smoking physicians [57].

As a positive finding, most smokers (90.6%) were serious about quitting smoking in the next few months, which reinforces the need to assist students in smoking cessation in schools for future healthcare workers [58].

The prevalence of risky drinking found in this sample (33%) was in line with that reported in other research among Norwegian students (35.9%) [59], although lower than those found, for example, among French [9] and Brazilian [60] medical students (47% and 53%, respectively). These differences, according to the country analyzed, coincide with those published in the general population in the Global Burden of Disease Study 2019 [61], with lower results in countries such as Spain and Norway (1.7%) and higher ones in countries such as France and Brazil (2.24 and 2.96%, respectively), which corroborates that the pattern of alcohol consumption in university students is strongly influenced by their sociocultural context [40].

Binge drinking is associated with multiple negative health consequences, such as an increased development of alcohol dependence in adulthood [62], among others. Despite this, it is highly frequent among young people, and especially college students, compared to the general population [62]. Proof of this is that 21.8% of our university sample carried out this practice at least monthly, quadrupling those found in the general national population over 15 years of age (6%), according to European surveys [47].

In this research study, the most used drug type during the last 30 days was cannabis, with a prevalence of 15.6%, similar to studies such as the one conducted by Reis et al. [8] in Brazilian students. The rest of the drugs were also used at a similar frequency, except for benzodiazepines, which were used more frequently (11.1%) than in similar populations in other countries (4–7.9%) [8]. This could be justified by the fact that Spain currently occupies the first place in benzodiazepine use in the world, according to the United Nations [63].

During COVID-19 confinement, modifications in toxic habits were objectified among college students, probably related to social isolation, limited access to harmful substances, and health-related reasons [38]. In that first stage of the pandemic, reductions in alcohol consumption were found to be between 21.6% and 28% in higher education students [38,39]. Although this study was conducted in a later period, when some restrictions had been reduced, 22.4% of students reported a decrease in alcohol consumption, which is consistent with a Norwegian study conducted over a similar period [59]. According to these results, alcohol consumption among university students seems to have decreased concerning the pre-pandemic period, and this effect is still maintained in the second year of the pandemic.

A national survey of the general Spanish population comparing tobacco, alcohol, and illicit drug use between the pre-pandemic period and the first year of COVID-19 showed a decrease in the use of all the drugs analyzed, especially in the 20–24-year age group, which may be due to the recreational use that often justifies their use at this age [64]. These reductions in toxic habits were also reflected in our results, except for smoking, which showed a slight net increase among the students in the sample.

When analyzing the relationships among between toxic habits, binge drinking and hazardous alcohol use formed with tobacco and cannabis use a dangerous combination triangle of addictive substances, which was also found in previous studies [7,52]. In turn, binge drinking at least once a month was associated with twice as much cocaine use. This association, documented before among medical students as high-risk drinkers [60], corroborates the need for a comprehensive preventive approach against the use of psychoactive substances in healthcare universities [9].

#### 4.1.2. Well-Being Measures

The study of stress in the university population is essential, due to the greater susceptibility it generates to mental health problems [18]. Perceived stress has been widely represented in this population, with high prevalence in the pre-pandemic era (6.3–13.3%) [18,19,20]. The transition from adolescence to adulthood and academic burden, among other factors, is linked to stress as a possible trigger [18]. The onset of the COVID-19 pandemic, marked by the confinement of the population, substantially increased severe stress among these youth to rates between 35.5 and 60.9% [32,33]. In addition to the existing causes, together with the social isolation already mentioned, there were also concerns about the continuation of the academic year, mobility restrictions, fear of COVID-19 disease, and in the case of healthcare students, also the interruption of clinical practice or frustration at not being able to contribute to health care at the beginning of the pandemic [65]. In this study, conducted in the second phase of the pandemic, almost half of the students surveyed reported increased stress, and 28.2% reported severe levels of stress, which is similar (16.5%) to the results reported by Zheng et al. [66] in a similar period and using the PSS-10. In a general analysis, a decrease in severe stress was observed with respect to the beginning of the pandemic, although the figures were still higher than those of the studies conducted before COVID-19; thus, we believe that it is necessary to continue monitoring this measure of well-being in this population.

The increase in perceived stress was accompanied by a reduction in sleep in almost half of the sample, which was congruent with the direct relationship that was objectified between the two factors in this and other investigations [32,38,67].

One-third of the students surveyed reported a fair, bad, or very bad health status, which was three times the prevalence found by Bennasar-Veny et al. [21] in another Spanish university in the year before the pandemic. The relationship found between higher levels of stress and poorer health may have led to these differences, although we could not confirm this, since the samples were different.

Instructing healthcare students in stress management could result not only in improving their sleep quality and health status, but also in helping them to acquire greater competence to provide safe and quality clinical care in the future [9]. This would serve them in their regular work as well as in extreme situations, such as probable future pandemics, wars, humanitarian catastrophes, etc., where the mental health of healthcare workers is at greater risk, due to the complexity of their work [29,30].

### 4.2. Relationships between Toxic Habits and Wellness Measures

Some studies have associated the university population with the consumption of harmful substances, such as tobacco and alcohol, with a higher level of stress [6,19,21].

However, in line with other investigations [7,22,23], among our results, we did not find a relationship between substance use and stress in this population. This could be because their motivations to consume harmful substances were more related to a recreational purpose than to alleviate their emotional distress, as was found by Gignon et al. [9] in a sample of medical students, among whom high-risk drinkers admitted doing so mainly for social reasons.

In contrast, smoking, both active and passive, in the student’s home was specifically linked to poorer perceived health status and shorter sleep duration. This was found by Altun [26], who observed that this factor had the greatest influence on a poorer sleep experience in a sample of university students. The dissemination among college students of these immediate negative consequences of smoking as well as the increase in smoke-free spaces should be established in all universities.

### 4.3. Gender Differences in Toxic Habits and Measures of Well-Being

There were gender differences, as female students presented a significant increase of 12.9% in risky drinking compared with males. This represents a singularity with respect to the rest of the studies consulted in the university population, where the male sex was a risk factor among risky drinkers both in the period before COVID-19 [6] and during the pandemic [59]. However, these data were congruent with results published by national agencies during the first year of the pandemic, which showed a 3% difference in favor of the female sex among young people in the general population in terms of consumption risk measured using the AUDIT-C [64]. In addition, studies based on large-population surveys found that among females, the trend toward risky drinking patterns was increasing [68]. It is important to highlight that the determination of this pattern makes a distinction between both sexes, lowering the threshold for the female sex, due to biological reasons; thus, an identical absolute consumption between men and women would result in a worse outcome among females. In contrast, we found no gender differences in the binge drinking pattern, for which we used a definition that does not distinguish between sexes.

In contrast to alcohol consumption risk, the female population consumed fewer cannabinoids (RR, 0.60; 95% CI (0.42–0.85); *p* < 0.001) than males, which was consistent with the reviewed studies [69].

Graves et al. [20] reported that university women presented different coping strategies to manage stressful situations with respect to their male counterparts, with more emotion-focused methods. This discrepancy could be linked to the higher rates of perceived stress found in the female population in this research study, which was widely documented in college students before the pandemic [20,21,22]. This study complemented other studies, which highlighted that these gender differences were maintained during the pandemic [32], specifically among university students in the field of healthcare [65,67].

In line with the relationship detected between higher levels of stress and a worse index of self-perceived health and insufficient sleep duration, we found that gender differences in stress also held true for the other measures of well-being. Gender differences in these health indicators were reported by Benham et al. [15] in a sample of university students analyzed in the period before the pandemic, and such indicators were still valid in this study.

Future research is required to continue to monitor how toxic habits and wellness measures will evolve in the college population once all the constraints brought on by the COVID-19 pandemic are removed.

### 4.4. Limitations

For future research, conducting random sampling and following up on the population with a post-survey could improve the quality of the study.

As this was a cross-sectional study, we could not establish causal relationships. Moreover, in this regard, variables such as motivations for consuming toxic substances or the causes of the worsening of well-being measures could have been evaluated, which would have allowed us to analyze gender differences in greater depth. However, as this was a voluntary, self-administered survey, we believe that a longer survey could have led to incomplete surveys. On the other hand, the students had the presence of a researcher while answering the survey, which could have improved the quality of the answers.

The use of a self-reported questionnaire as a measurement instrument might have underestimated substance use due to social desirability bias, although these instruments are valid in a university population for analyzing substance use [70]. In addition, since the data were obtained through a survey carried out by the students who attended class, we lacked data on students who did not usually go to class, which could be a selection bias because school absenteeism can sometimes be related to the worst health habits.

Regarding the investigation of perceived changes from the period before the COVID-19 pandemic, this study was based on students’ retrospective recall, which could have been affected by recall bias [71]; thus, these findings must be considered as perceptions of the individual.

Finally, the participants were mostly women (66.3%) compared with men (33.7%), with proportions similar to those reported in the different studies carried out in university populations [21].

## 5. Conclusions

This study revealed a considerable presence of toxic habits, severe perceived stress, and insufficient sleep duration among Spanish healthcare students with a perceived worsening with the COVID-19 pandemic, which highlights the need to implement specific public health educational strategies in the universities of future health providers.

The association found between smoking, harmful drinking patterns, and the consumption of cannabinoids implies recommending that these preventive programs treat all psychoactive substances simultaneously.

Since severe levels of stress were associated with poorer perceived health status and shorter sleep duration, and the latter two measures with smoking, we suggest that interventions should address multiple behaviors by focusing on stress management, education about habits of sleep, and the immediate consequences of unhealthy habits as pillars to improve the health of students.

Worse results were found among female students in all the well-being measures analyzed and in risky alcohol consumption, which leads us to propose that these interventions be carried out with a gender perspective.

## Figures and Tables

**Table 1:** Academic and socioeconomic characteristics among the participants (*n* = 982).

Academic Major	*n* (%)
Biomedicine	72 (7.3%)
Sports sciences	76 (7.8%)
Nursing	356 (36.3%)
Physical therapy	94 (9.6%)
Biomedical engineering	17 (1.7%)
Medicine	367 (37.3%)
**Year**	***n* (%)**
1	427 (43.5%)
2	346 (35.2%)
3	91 (9.3%)
4	60 (6.1%)
5	32 (3.3%)
6	26 (2.6%)
**Coexistence during the Academic Year**	***n* (%)**
Living alone	66 (6.7%)
In the family home	416 (42.3%)
In a shared flat	273 (27.9%)
In a student residence	227 (23.1%)
**Economic Support for University Costs**	***n* (%)**
Family	865 (88.1%)
Grants	57 (5.8%)
Student’s job	60 (6.1%)

**Table 2:** Frequency of binge drinking and last 30 days’ use by drug type (*n* = 982).

	Daily *n* (%)	Weekly *n* (%)	Monthly *n* (%)	<Once a Month *n* (%)	Never *n* (%)
Binge drinking	1 (0.1%)	75 (7.6%)	138 (14.1%)	283 (28.8%)	485 (49.4%)
	**Daily** ***n* (%)**	**Weekly** ***n* (%)**	**Occasional** ***n* (%)**		**Never** ***n* (%)**
Benzodiazepines	42 (4.3%)	13 (1.3%)	54 (5.5%)		873 (88.9%)
Cannabinoids	39 (4%)	27 (2.7%)	87 (8.9%)		829 (84.4%)
Cocaine	0	5 (0.5%)	4 (0.4%)		973 (99.1%)
Ecstasy	0	3 (0.3%)	10 (1%)		969 (98.7%)
Amphetamines	0	2 (0.2%)	7 (0.7%)		973 (99.1%)
LSD	0	1 (0.1%)	2 (0.2%)		979 (99.7%)

**Table 3:** Health status (*n* = 982).

Health Status	*n* (%)
Very good	162 (16.5%)
Good	533 (54.3%)
Fair	246 (25%)
Bad	35 (3.6%)
Very bad	6 (0.6%)

**Table 4:** Quantification of stress perception, according to the PSS-10 questionnaire, between males and females.

Stress Level	Males, *n* (%) (*n* = 330)	Females, *n* (%) (*n* = 652)	*p*
Low	42 (12.7%)	71 (10.8%)	0.004
Medium	217 (65.7%)	377 (57.8%)	
High	71 (21.6%)	204 (31.4%)	

**Table 5:** Distribution of smokers and non-smokers among academic majors (*n* = 982).

Academic Majors	Active Smokers *n* (%)	Non-Smokers *n* (%)	*p*
Biomedicine	18 (25%)	54 (75%)	<0.001
Sports sciences	17 (22.4%)	59 (77.6%)	
Nursing	146 (40.8%)	210 (59.2%)	
Physical therapy	25 (26.6%)	69 (73.4%)	
Biomedical engineering	3 (17.6%)	14 (82.4%)	
Medicine	96 (26.2%)	271 (73.8%)	

**Table 6:** Students’ perception of the influence of the COVID-19 pandemic on their lifestyles (*n* = 982).

	Increased Habit/Worsening *n* (%)	Similar Habit *n* (%)	Reduced Habit/Improvement *n* (%)	Net Effect (%)
Smoking	118 (12%)	759 (77.2%)	105 (10.8%)	1.2% worse
Alcohol consumption	222 (22.6%)	540 (55%)	220 (22.4%)	0.2% better
Illicit drugs	45 (4.6%)	891 (90.7%)	46 (4.7%)	0.1% better
Stress perception	484 (49.3%)	272 (27.7%)	226 (23%)	26.3% worse
Sleep habit	434 (44.2%)	404 (41.2%)	144 (14.6%)	29.6% worse
Health status	272 (27.7%)	410 (41.8%)	300 (30.5%)	2.8% better

## Data Availability

The datasets used and/or analyzed during the current study are available from the corresponding author upon reasonable request.
